# Training for clinical research professionals: Focusing on effectiveness and utility

**DOI:** 10.4103/2229-3485.71767

**Published:** 2010

**Authors:** Viraj Rajadhyaksha

**Affiliations:** *Associate Director-Medical Liaison, Sanofi-Aventis, Mumbai, India*

The role of effective training in professional development is very significant. In a highly specialized field such as clinical research, gaining of adequate knowledge, attaining practical skills and developing the right attitude are imperative.[1] Equally important is to define the various levels of training required for different functional levels of clinical research professionals. At a clinical research associate level, the knowledge of Indian GCP guidelines, applicable regulations such as Schedule Y, and interpersonal skills with site teams are essential. On the other hand, at a team leader level, working with different individuals and people management skills will be required. The head of a clinical research group will require additional skills such as business acumen to expand the operations to the next level in line with guidelines and regulations. A survey in 2008 identified knowledge on ethics, informed consent, and audits and drug development process as crucial for investigators’ sites. Skills such as teamwork, negotiation and interpersonal skills were also highlighted as the most important.[2] The needs of pharmaceutical physicians can be broader than all that mentioned above.

Realizing that effective training is important for the growth of clinical trials in India, several training programs have been initiated since 2002. Some of these are attached to private, deemed or even public universities. Some are courses actually conducted by universities. While many of these courses started for clinical research associates (CRA) and study managers, they have also been expanded to areas such as data management, medical writing and pharmacovigilance. Based on our experience, many of the individuals who complete these courses have a fair understanding of the knowledge domain but less development of the skills domain due to limited practical exposure. As a result, extensive in-house training is required to improve their efficiency in day-to-day working. This obviously results in an increased time lag before individuals can start working independently with minimum oversight.

In this issue of the Journal, two articles addressing the training issue have been published. The first one is a survey which looks at various domains essential for a CRA and enlists those areas or topics which have been reported as basic essential topics for effective functioning of a CRA.[3] The second is a review article based on real life experience, mentioning challenges in current training programs, problems faced by employers as well as new employees, and some suggestions to address these problems.[4]

To make training more effective, we believe that we must concentrate on two aspects of the training activity: Quality and Target Audience.

## QUALITY

In the absence of an overall guidance/regulatory body, there is a lack of harmonization in the training programs imparted by different organizations. While the broad areas seem to be similar, the actual information given under each can vary significantly. Secondly, while the knowledge domain is often effectively addressed to a reasonable extent through classroom teaching programs, exposure to the skills domain, especially soft skills and interpersonal skills, can be often inadequate. Right attempts to develop the attitude domain are hardly a part of the core curriculum.

### Lack of harmonization

There is a requirement to develop uniform requirements of training programs with specifics, in terms of time spent and exposure on knowledge and skills. We need to institutionalize a formal dialog between various training institutes to improve the quality of training and also create a cross-functional group of industry–academia professionals to develop common curricula.

### Low focus on skill development

Skills required at different levels may be identified by conducting more surveys and research. As part of training programs, faculty must focus on skill development through workshops and role-plays. To gain some hands-on experience, training institutes can collaborate with industry on development of summer jobs for interns.

### Lack of differentiation in training

To improve the efficiency of a training program, we must differentiate requirement at basic and proficiency levels, concentrating on knowledge in the former and skills/attitude in latter. Instead of running several general programs, we must develop more specialized programs, e.g., advanced monitoring, study management, quality, pharmacovigilance at proficiency level by differentiating requirements of end-users [e.g., the Association of Clinical Research Professionals (ACRP) develops and conducts programs for audiences at different proficiency levels by classroom, webinars, etc].[5]

## TARGET AUDIENCE

On one hand while a number of programs help in increasing the number of potential CRAs and project managers (sponsor’s team), on the other hand, there is limited organized work done on the investigator/site and ethics committee (EC) front. The lack of adequately trained investigator sites is the major hurdle in way of expansion of clinical trials in India. In addition, there are some functions, such as pharmaceutical physicians, who also do not feature in any training program. We propose the following measures to effectively target all stakeholders in clinical research.

### Investigator and site teams

We must develop investigator specific programs in collaboration with medical colleges and universities and include modules on research – clinical trials, epidemiology in undergraduate medical curricula. To develop the skill domain, we propose to include practical exposure to at least one form of prospective research during internship. The Korean Network of Clinical trials (KONECT) program is a good example of structured programs toward clinical pharmacology, clinical research coordinators, etc.[6]

### Ethics committees

Formal sharing of experiences between ECs is necessary. To make the entire training process more accessible, online training modules mandatory for all EC members can be developed.

### Pharmaceutical physicians

As the specialty of pharmaceutical medicine develops in India, we must create a regular forum for exchange of information amongst pharmaceutical physicians and develop specialized pharmaceutical medicine program for experienced physicians in line with the suggestions by the Faculty of Pharmaceutical Medicine’s Pharmaceutical Medicine Specialty training program.[7]

As a clinical research community, we propose that we prioritize some specific aspects. Firstly, we focus on improving the quality of training programs by defining end-user need and then customizing training activities to fit those needs. Secondly, we differentiate needs at entry level and for experienced professionals, and implement programs specifically addressing individuals at different levels. Thirdly, industry–academia collaboration is mandatory for conducting more programs toward investigators and sites to improve research infrastructure in hospitals and private clinics. Fourthly, we believe that our focus has to be on competency building and not only on capacity building as has been the focus for sometime.

As a first step toward harmonization, the Training council of the Society announces to take the lead in launching the first accreditation program. We propose to launch the program by early 2011 for entry level clinical research professionals. The examination would evaluate a candidate’s understanding of current regulations, guidelines and ability to identify responsibilities of various stakeholders in clinical trials.

In our country where knowledge is worshipped, it is the responsibility of professionals to develop and implement training programs for those who will walk on this path in the future. If we do not stand up to this responsibility, we would fail in our duty to create the next generation of professionals who are likely to take clinical research in specific and India in general to the next level. If this does not happen for some reason, we alone would be responsible for the blame.

## DISCLAIMER

The article has been written in the author’s personal capacity. Views and opinions expressed are those of the author and may not necessarily be those of sanofi-aventis.
